# Linkage and association of variants in the dopamine receptor 2 gene (*DRD2*) with polycystic ovary syndrome

**DOI:** 10.1186/s13048-023-01205-2

**Published:** 2023-08-10

**Authors:** Mutaz Amin, Nicholas Horst, Claudia Gragnoli

**Affiliations:** 1 INSERM, US14-Orphanet, Paris, 75014 France; 2 Creighton University School of Medicine, Omaha, NE 68124 USA; 3 Division of Endocrinology, Department of Medicine, Creighton University School of Medicine, Omaha, NE 68124 USA; 4 Department of Public Health Sciences, Penn State College of Medicine, Hershey, PA 17033 USA; 5 Molecular Biology Laboratory, Bios Biotech Multi-Diagnostic Health Center, Rome, 00197 Italy

**Keywords:** Polycystic ovarian syndrome, PCOS, Dopamine receptor 2, *DRD2*, Gene, Prolactin receptor, Type 2 diabetes, Depression, Association, Infertility

## Abstract

Polycystic ovarian syndrome (PCOS) is a disorder with a foundation of neuroendocrine dysfunction, characterized by increased gonadotropin-releasing hormone (GnRH) pulsatility, which is antagonized by dopamine. The dopamine receptor 2 (DRD2), encoded by the *DRD2* gene, has been shown to mediate dopamine’s inhibition of GnRH neuron excitability through pre- and post-synaptic interactions in murine models. Further, DRD2 is known to mediate prolactin (PRL) inhibition by dopamine, and high blood level of PRL have been found in more than one third of women with PCOS. We recently identified *PRL* as a gene contributing to PCOS risk and reported *DRD2* conferring risk for type 2 diabetes and depression, which can both coexist with PCOS. Given DRD2 mediating dopamine’s action on neuroendocrine profiles and association with metabolic-mental states related to PCOS, polymorphisms in *DRD2* may predispose to development of PCOS. Therefore, we aimed to investigate whether *DRD2* variants are in linkage to and/or linkage disequilibrium (i.e., linkage and association) with PCOS in Italian families. In 212 Italian families, we tested 22 variants within the *DRD2* gene for linkage and linkage disequilibrium with PCOS. We identified five novel variants significantly linked to the risk of PCOS. This is the first study to identify *DRD2* as a risk gene in PCOS, however, functional studies are needed to confirm these results.

**Supplementary Information:**

The online version contains supplementary material available at 10.1186/s13048-023-01205-2.

## Introduction

Polycystic ovarian syndrome (PCOS) is a common endocrinopathy of reproductive age women with a worldwide prevalence of 5–10% [1]. It is classically defined by oligo-/anovulation, hyperandrogenism, and formation of peripheral antral follicles in the ovaries, and it is associated with infertility, insulin resistance, type 2 diabetes, and depression [2–6]. PCOS is a disorder with a foundation of neuroendocrine dysfunction, characterized by increased gonadotropin-releasing hormone (GnRH) pulsatility and luteinizing hormone (LH) and follicle-stimulating hormone (FSH) ratio [7]. The pathogenesis of PCOS, however, is multifaceted and heterogenous, rooted in complex environmental and genetic etiology not yet fully defined, although clustering and susceptibility loci demonstrate a genetic component [8–10].

Reproductive function is primarily driven by the hypothalamic-pituitary-gonadal (HPG) axis [11]. Pulsatile GnRH secretion by the hypothalamus induces LH and FSH release by the anterior pituitary [11]. The HPG axis is regulated by numerous hormones, peptides, and neurotransmitters [11]. Aberrations in these regulating components thereby may induce endocrinopathies such as PCOS. Ovarian thecal and stromal cell hyperplasia and hyperandrogenism has been suggested to be a consequence of LH surplus sustained by rapid GnRH pulse frequency [12]. Of note, dopamine is antagonistic to GnRH release and pulsatility [13–15]. Also, prolactin (PRL) blood levels if elevated can suppress GnRH, and women with PCOS have been found having high PRL blood levels [16]. Dopamine levels are higher in patients with PCOS [17], and dopamine infusions have been shown to induce a significant reduction in LH levels in humans, and bromocriptine, a dopamine agonist, has demonstrated efficacy in reducing LH:FSH ratios and restoring ovarian function in women with PCOS [18, 19]. Dopamine exerts its effects through dopamine receptors expressed in the brain and peripheral tissues [20].

The dopamine receptor 2 (DRD2), encoded by the *DRD2* gene, has been shown to mediate dopamine’s inhibition of GnRH neuron excitability through pre- and post-synaptic interactions in murine models [14] and to suppress PRL secretion [21]. Thus, if *DRD2* carries genetic variants affecting its function, it can contribute to higher PRL levels inhibiting the reproductive cycles and/or contribute to GnRH abnormal pulsatility. Studies in ewe models have also suggested that DRD2 affects hypothalamic *GnRH* gene expression, and DRD2 specific antagonists stimulate GnRH and LH pulsatility [22]. Related to this, lowered brain dopamine levels and reduced DRD2 expression have been found in PCOS rat models [23]. DRD2 may also play a role in metabolic phenotypes associated with PCOS. Studies have demonstrated that reduced DRD2 activation is involved in development of insulin resistance in obese mice, and pharmaceutical agonism of DRD2 alleviates insulin resistance in animals [24, 25]. Importantly, insulin resistance is considered an important associated feature of hyperandrogenic anovulation in PCOS [26, 27]. Reduction in dopaminergic tone, as well as DRD2 signaling and regulation is also implicated in follicles of PCOS ovaries from rat PCOS models [28].

Given DRD2-mediated dopamine inhibition of PRL [21], and the elevated PRL blood levels found in PCOS [16], we studied the PRL pathway in PCOS and recently identified the prolactin receptor (*PRLR*) gene as contributing to PCOS risk [29]. Of note, we also reported *DRD2* [30] and *PRL*-variants [31] conferring risk for type 2 diabetes and depression, which can both coexist with PCOS [6, 32]. Given dopamine’s action through DRD2 in neuroendocrine profiles and association with metabolic-mental states related to PCOS, polymorphisms in *DRD2* may predispose to development of PCOS. Therefore, we aimed to investigate whether *DRD2* variants confer risk to PCOS in Italian families.

## Materials and methods

Among 212 Italian families with rich type 2 diabetes (T2D) family history, phenotyped for PCOS per Rotterdam diagnostic criteria [33]. cases were selected if at least two of the following were present: chronic anovulation or oligomenorrhea, clinical or biochemical hyperandrogenism, and/or polycystic ovaries [33]. We amplified 22 microarray-based single nucleotide polymorphisms (SNPs) located within the *DRD2* gene (Supplementary Table 1). We excluded genotyping and Mendelian errors using PLINK [34]. We then analyzed the 22 SNPs for 2-point parametric-linkage to and linkage-disequilibrium (LD, i.e., linkage and association) with PCOS across the following models: dominant completely penetrant (D1), dominant incompletely penetrant (D2), recessive completely penetrant (R1) and recessive incompletely penetrant (R2). The linkage and LD analysis were performed using Pseudomarker [35]. We inferred the presence or absence of LD blocks by calculating the correlation coefficient between variants using the data from the 1000 Genome project (https://www.internationalgenome.org/data-portal/population/TSI). The study was institutionally approved by the Bios Ethical Committee.

## Results

**Fig. 1:**
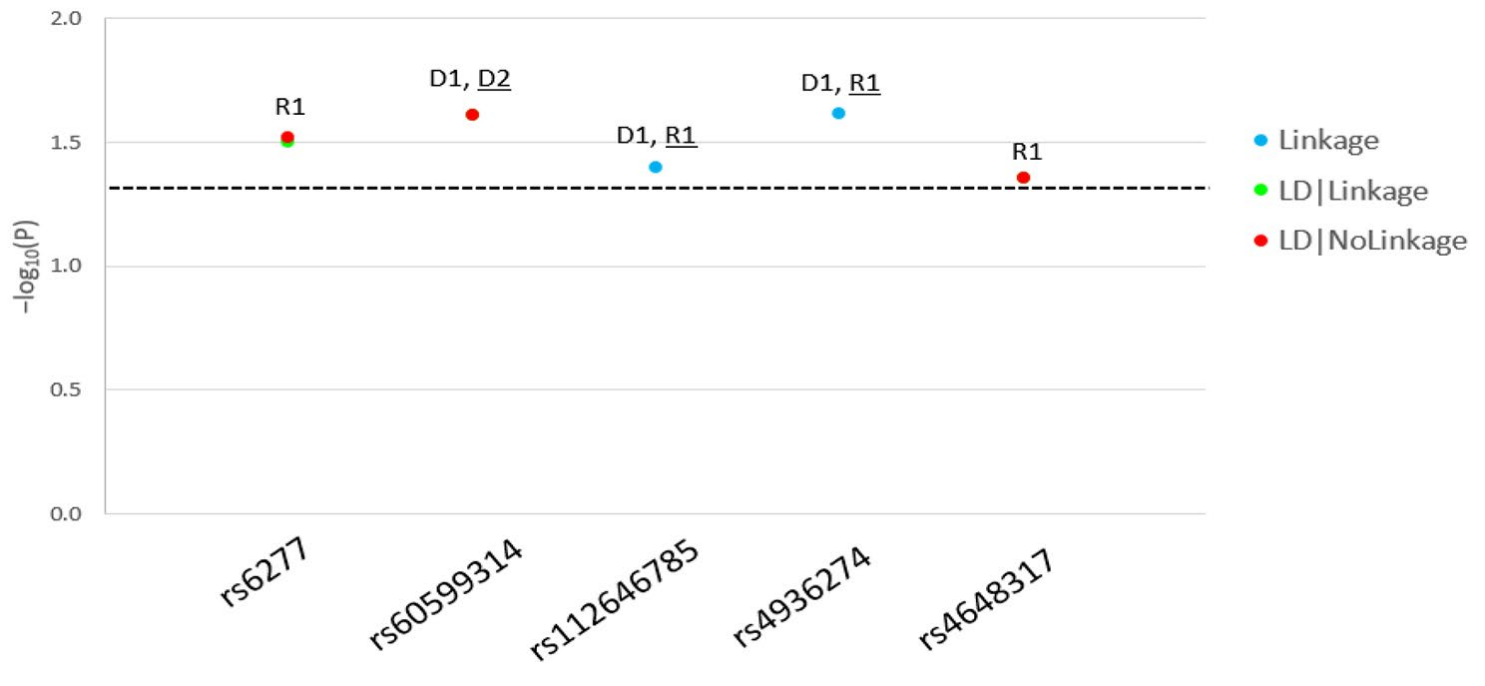
Parametric analysis results of *DRD2*-risk single nucleotide polymorphisms (SNPs) in polycystic ovarian syndrome (PCOS) **Legend.** For each *DRD2*-risk SNPs in PCOS, we present the − log10(P) as a function of the significant (*p* < 0.05) test statistics [(Linkage, linkage disequilibrium (LD)|Linkage, LD|NoLinkage] and per inheritance model. D1: dominant, complete penetrance, D2: dominant, incomplete penetrance, R1: recessive, complete penetrance, R2: recessive, incomplete penetrance

We found a total of 5 variants (rs6277, rs60599314, rs112646785, rs4936274, rs4648317) significantly linked to and/or in LD with PCOS (Table 1). Linkage and association (LD) were statistically significant across different inheritance models (Fig. 1). None of the 5 variants had been previously reported with PCOS.

**Table 1:** Polycystic ovarian syndrome (PCOS) *DRD2*-risk single nucleotide polymorphisms (SNPs)

Model^1^	SNP	Position	Ref	Alt	Risk Allele	Consequence	LD block	Reported in PCOS or related phenotype? ^2^
R1	rs6277	113,412,737	G	A	A	Synonymous (P319P)	Independent	Obesity [36]
D1, D2	rs60599314	113,435,709	C	T	T	Intronic	Independent	Novel
D1, R1	rs112646785	113,444,554	T	C	T	Intronic	Independent	T2D-MDD [30]
D1, R1	rs4936274	113,459,729	A	G	A	Intronic	Independent	Novel
R1	rs4648317	113,460,810	G	A	A	Intronic	Independent	Novel

^1^Models: D1: dominant, complete penetrance, D2: dominant, incomplete penetrance, R1: recessive, complete penetrance, R2: recessive, incomplete penetrance, T2D: type 2 diabetes, MDD: major depressive disorder; ^**2**^PCOS-related phenotypes: type 2 diabetes, obesity, insulin resistance, metabolic syndrome, hyperglycemia, oligoamenorrhea, anovulation, irregular menses, hyperandrogenism, male-pattern baldness, acne, hirsutism, infertility

### *In-silico* analysis

We analyzed the *DRD2*-risk variants by different bioinformatics tools that predict their functional role in transcription factor (TF) binding (SNP Function Prediction) [37], miRNA binding (mirSNP) [38], splicing (SpliceAI) [39], and regulatory potential (RegulomeDB) [40]. We found that the risk allele (T) of the variant rs60599314 disrupts the binding of transcription factor AP-2-alpha (Tfap2a) whose upregulation impairs meiosis in mouse oocytes [41].

## Discussion

PCOS is a complexly inherited disorder, and variations in several neuroendocrine-related genes were shown to mediate a risk or susceptibility [42]. In this study, we have shown for the first time that the *DRD2* gene is also a potential risk gene in PCOS. We have recently reported the implication of *DRD2* gene in the risk of T2D and depression in the same multigenerational Italian families under study [30]. The same risk allele (T) of the variant rs112646785 was previously linked to the risk of T2D and MDD comorbidity [30] and now to PCOS, confirming the closely intertwined genetic and phenotypic relationships between these complex disorders [6, 32] as well as the possible pleiotropic role of *DRD2*. None of the five *DRD2*-variants reported in our study have been linked before to the risk of PCOS and therefore are novel. Some risk variants in our study, however, have been previously studied and/or reported with PCOS-related phenotypes. The risk allele (A) of the variant rs6277 was associated with obesity [36]. The same variant has also been studied with endometriosis-associated infertility (along with rs4648317) [43] and insulin resistance and T2D [44], but no association has been found. The non-risk allele (C) of this variant rs6277, however, correlated with hyperglycemia in schizophrenic patients, indicating the presence of possible LD with other undetected contributing variants [45].

The roles played by *DRD2*-risk variants in the pathogenesis of PCOS have yet to be defined. The effect could be mediated by alteration of TF binding as predicted by our *in-silico* analysis. The risk allele (T) of the variant rs60599314 was predicted to disrupt the binding of transcription factor AP-2-alpha (Tfap2a), whose upregulation impairs meiosis in mouse oocytes [41]. The pathogenic role could also be mediated by alteration of DRD2 properties. The risk allele (A) of the variant rs6277 was previously associated with increased receptor density and affinity in the striatum [46] which if confirmed at the level of the hypothalamus might mediate the DRD2 effects on GnRH pulsatility in PCOS. However, if the DRD2 density and affinity were to be constitutionally present, the effect would not account for the higher peripheral dopamine levels reported in patients with PCOS by a possible DRD2 resistance [17]. Given that elevated blood levels of PRL were found in more than one third of PCOS women [16], and due to our previous findings of PRL contributing to T2D and depression [31], both phenotypes associated with PCOS [6, 32], it is also possible that some DRD2 variants might impair PRL secretion regulation and indirectly impair gonadotropin secretion and reproductive cycles. Given the complex heterogeneity of PCOS, more than one molecular genetic pattern can contribute to it, and PCOS subjects may be predisposed to one molecular distinct entity vs. another, or present with overlapping molecular underpinnings. Therefore, functional studies are still needed to confirm and explain these results. It is also important to replicate the genetic results in other ethnic groups.

### Electronic supplementary material

Below is the link to the electronic supplementary material.

Supplementary Material 1

Supplementary Material 2

## Data Availability

The study data are available on reasonable request, and due to lacking specific patients’ consent and privacy restrictions, they are not publicly available.

## Declarations

### Authors’ information

C.G. is Professor of Medicine and Chief of Endocrinology and Endowed Puller Chair at Creighton University School of Medicine, Omaha, NE, and Adjunct Professor of Public Health Sciences, Penn State University College of Medicine, Hershey, PA; N.H. is a medical student at Creighton University School of Medicine, Omaha, NE; M.A. has an MD and a PhD in Genetics (University of Paris) and is a geneticist at Orphanet | INSERM-US14, Paris, France, and member of the executive committee, European Reference Network for the Intellectual Disabilities (ERN-ITHACA) and the International Consortium of Gene Curation Coalition (GenCC).
