# Supplementary material for: Linkage and association of variants in the dopamine receptor 2 gene (DRD2) with polycystic ovary syndrome
Source: J Ovarian Res. 2023 Aug 10;16:158. doi: 10.1186/s13048-023-01205-2 (PMC10416464; doi:10.1186/s13048-023-01205-2)
Supplement: Supplementary file 2 — Supplementary Material 2 [file 13048_2023_1205_MOESM2_ESM.docx]

| **Supplementary Table 1** | | | | |
| --- | --- | --- | --- | --- |
| **The 22 amplified microarray-based single nucleotide polymorphisms located within the *DRD2* gene** | | | | |
| **CHR** | **Position** | **rsID** | **REF** | **ALT** |
| 11 | 113410675 | rs6276 | C | T |
| 11 | 113412737 | rs6277 | G | A |
| 11 | 113412766 | NA | G | A |
| 11 | 113412966 | rs1076560 | C | A |
| 11 | 113415071 | rs35608204 | A | G |
| 11 | 113416972 | rs1800499 | C | T |
| 11 | 113425552 | rs1079598 | A | G |
| 11 | 113425897 | rs1079596 | C | T |
| 11 | 113426463 | rs1125394 | T | C |
| 11 | 113428395 | rs34535530 | G | A |
| 11 | 113428551 | rs75349786 | A | G |
| 11 | 113435709 | rs60599314 | C | T |
| 11 | 113436043 | rs4436578 | C | T |
| 11 | 113439147 | rs11214606 | C | T |
| 11 | 113444554 | rs112646785 | T | C |
| 11 | 113447251 | rs4245146 | T | C |
| 11 | 113459729 | rs4936274 | A | G |
| 11 | 113460810 | rs4648317 | G | A |
| 11 | 113463484 | rs118168024 | A | G |
| 11 | 113463506 | rs34632468 | T |  |
| 11 | 113470861 | rs35659571 | C | G |
| 11 | 113474844 | rs117816894 | C | T |
| **Legend.** CHR = chromosome, REF = Reference alleles, ALT = Alternative allele. | | | | |
